# Using discrete choice experiment to investigate public preferences for osteoporosis community-level management strategies in China

**DOI:** 10.1007/s00774-025-01659-y

**Published:** 2025-11-21

**Authors:** Yijia Feng, Lu Jing, Luying Zhang

**Affiliations:** 1 School of Public Health, Fudan University, Shanghai, 200032 China; 2 Yangtze Delta Institute of Health Insurance Research, Fudan University, No. 130 Dongan Road, Shanghai, 200032 China

**Keywords:** Osteoporosis, Community-level management, Public preferences, Discrete choice experiment

## Abstract

**Introduction:**

Osteoporosis is highly prevalent in China and imposes a substantial economic burden. Early community-level management is pivotal and community health centers play an important role in prevention and management. As shared decision-making in medication expands, understanding public preferences can help improve community health services. This study investigated public preferences of osteoporosis community-level management in China, considering access to screening information, screening duration, service supplier, mode of administration, management approach and out-of-pocket costs.

**Materials and Methods:**

A discrete choice experiment (DCE) was conducted in Shanghai among community-dwelling adults. We constructed a mixed logit model with a total of 14 levels of the above 6 attributes. Willingness-to-pay (WTP) and scenario predictions were performed. Exploratory subgroup analyses assessed heterogeneity by age, income, geographic location and self-reported osteoporosis.

**Results:**

A total of 170 valid questionnaires were collected from 6 communities in Shanghai. OOP costs had the largest impact on utility, followed by screening duration, service supplier and access to screening information. Subgroup analyses revealed that shorter screening duration and specialist-provided screening services had greater positive impact on utility among suburban residents than among urban sample. Scenario predictions suggested that the combination of shorter screening duration, service supplied by tertiary-hospital specialists and management supplied by general practitioners meaningfully increased predicted participants’ utility.

**Conclusion:**

Public preferences favored lower costs, convenient screening, clear information and specialist-led screening with general-practitioner-delivered management. Understanding the impact of attributes in community-level management strategies on residents’ utility and willingness to pay is important for optimizing community-level management strategies.

**Supplementary Information:**

The online version contains supplementary material available at 10.1007/s00774-025-01659-y.

## Introduction

Osteoporosis, a chronic disease characterized by low bone mass, increased bone fragility, and susceptibility to fracture, produces an enormous economic burden of disease both globally and in China. According to data from the *Global Burden of Disease*, the *disability-adjusted life years (DALYs)* due to low bone mineral density reached 16,647,466.4 years in 2019 [1]. As reported in China, the prevalence rate of osteoporosis was 32.1% in women and 6.0% in men over 50 years of age in 2018, with the number of fracture patients predicted to reach 5.99 million in 2050 [2].

Community health service centers (CHCs) in China play a pivotal role in the prevention and regular management of chronic diseases including osteoporosis. As the front units for chronic disease control, CHCs usually undertake the functions of health education and early screening, initial diagnosis and daily management of chronic diseases after diagnosis [3]. The community can intervene mainly in early screening for prevention and graded management of osteoporosis patients. The interventions encompass knowledge popularization seminar, screening for high-risk population during outpatient services and physical examinations, personalized services including medication, rehabilitation, diet and other lifestyle services for patients as well as psychological counseling support [4, 5]. The aim of interventions at community level is to achieve continuous and life-cycle disease management of osteoporosis, which helps to identify the population in high risk at an early stage and improve patients’ adherence to treatment strategies, ultimately reducing the prevalence of osteoporosis.

The concept of “patient-centered” in China calls for extension beyond the needs of patients to incorporate preferences of the general public [6, 7]. This perspective is particularly important at the community level, where interventions aim not only to manage osteoporosis among patients but also to prevent its onset in high-risk groups. Understanding how both patients and non-patients value sessions of intervention is essential for improving adherence and enhancing service quality of community programs. However, public preferences for osteoporosis still remain insufficiently addressed even though the importance of integrating that into health decision-making has been increasingly recognized [8].

Existing studies on osteoporosis community interventions have primarily focused on evaluating the intervention outcomes [9–11]. In addition to clinical indicators such as bone metabolism markers and 24-h urinary calcium values of osteoporosis patients, many have assessed residents’ changes in the cognitive level in osteoporosis, life behaviors, and preventive awareness before and after the intervention. In terms of intervention modalities, domestic and international studies have concentrated largely on osteoporosis drug treatment. For non-patients, common community strategies include health education, regular monitoring, lifestyle management, and early screening measures [12, 13] such as *Osteoporosis Self-Assessment Tool*, *International Osteoporosis Foundation’s one-minute risk test*, and lumbar spine X-rays. Most preference-related studies on osteoporosis focused on the treatment preferences of patients [14–20]. Attributes always included treatment efficacy [14, 15] (i.e., pain relief, improved mobility, and reduced risk of fractures), side effects [14, 16–18] (i.e., nausea), mode and frequency of administration [17, 18], total treatment duration [18], drug dosage [19] and out-of-pocket costs (OOP) [16–20]. Far less attention has been given to preferences of general public or a broader scope of community-level interventions.

Discrete choice experiment (DCE) is an important quantitative method, in which participants are usually presented with two or more hypothetical scenarios with the task of choosing one, and its scientific rationale has been widely validated in the health field [21–23]. This approach is well-suited to capture general public preferences onosteoporosis community interventions, thereby providing evidence to guide the design of more acceptable and effective community-based interventions.

The aim of this study was, therefore, to assess China’s public preferences for osteoporosis community interventions using a DCE. It also endeavored to identify the heterogeneity of participants’ preferences and their willingness to pay (WTP) for each attribute, further providing advice for improving health management of osteoporosis at the community level.

## Materials and Methods

### Ethics approval and consent to participate

Ethical approval for this study was approved by the Ethics Review Committee of the School of Public Health, Fudan University (IRB#2023- TYSQ-02-261) in accordance with the Declaration of Helsinki and CIOMS Guideline. The informed consent to participate was obtained from all of the participants in the study.

### Definition of community intervention

**Fig. 1:**
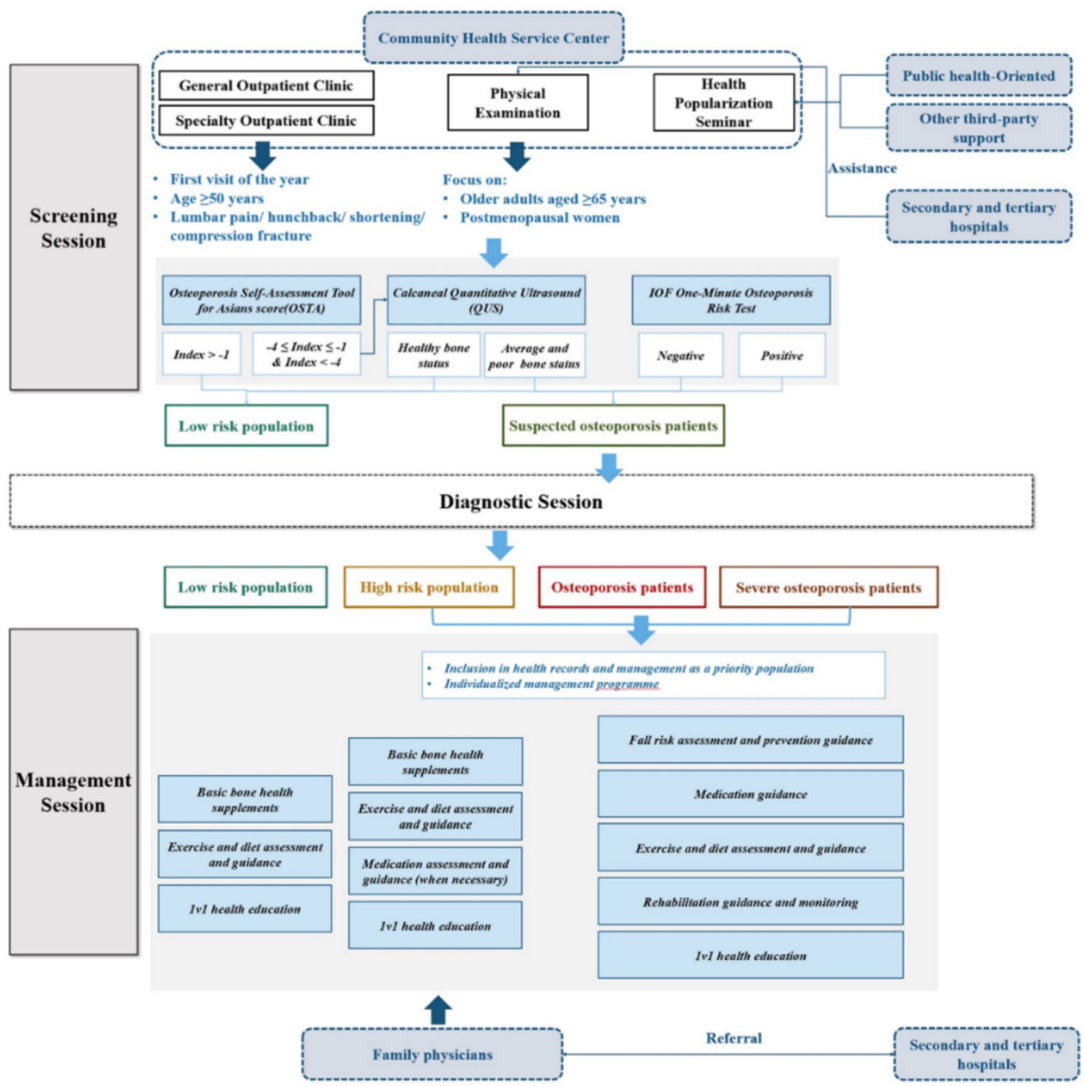
Definition of community intervention on osteoporosis

In our study, community intervention for osteoporosis was defined as comprising 2 core components: screening and management. As illustrated in Fig. 1, the screening session involves identifying individuals at risk through community-level activities, while the management session focuses on follow-up, lifestyle guidance, and targeted preventive measures. Diagnostic session, typically conducted in higher level facilities, is presented for completeness but not our primary focus.

### Attributes and levels

A total of 5 attributes were ascertained through the literature, including access to screening information, screening duration, service supplier, mode of administration, and OOP costs. The management approach included face-to-face consultations with experts, including 3 osteoporosis specialists from Shanghai’s tertiary hospitals, 3 community health service center managers, and 2 community general practitioners (GPs). The attributes and corresponding levels are detailed in Table 1.

**Table 1:** Attributes and levels for discrete choice experiment questions

Attributes	Definition of attributes	Levels
1. Access to screening information	Access to community screening information about osteoporosis	• When physical examination • From community outreach • From GP
2. Screening duration	Time taken from putting on screening device to finish the whole screening process	• Within 0.5 h • 0.5 to 1 h
3. Service supplier	Who will provide screening and treatment services	• Community GP • Specialists from tertiary hospitals (who come to the community to run sentinel clinics)
4. Mode of administration	The frequency and route by which medication enters the body	• Weekly oral • Quarterly or annual injection
5. Management approach	Supervisory and managerial approaches to the daily control of osteoporosis in patients	• Self-monitoring • Supervision of GP
6. Out-of-pocket costs	Individual expenses after reimbursement by the Shanghai basic medical insurance	• None • 500 Chinese *yuan* per year (*USD* 69.94) • 1000 Chinese *yuan* per year (*USD* 139.88)

### Questionnaire and DCE instrument design

**Fig. 2:**
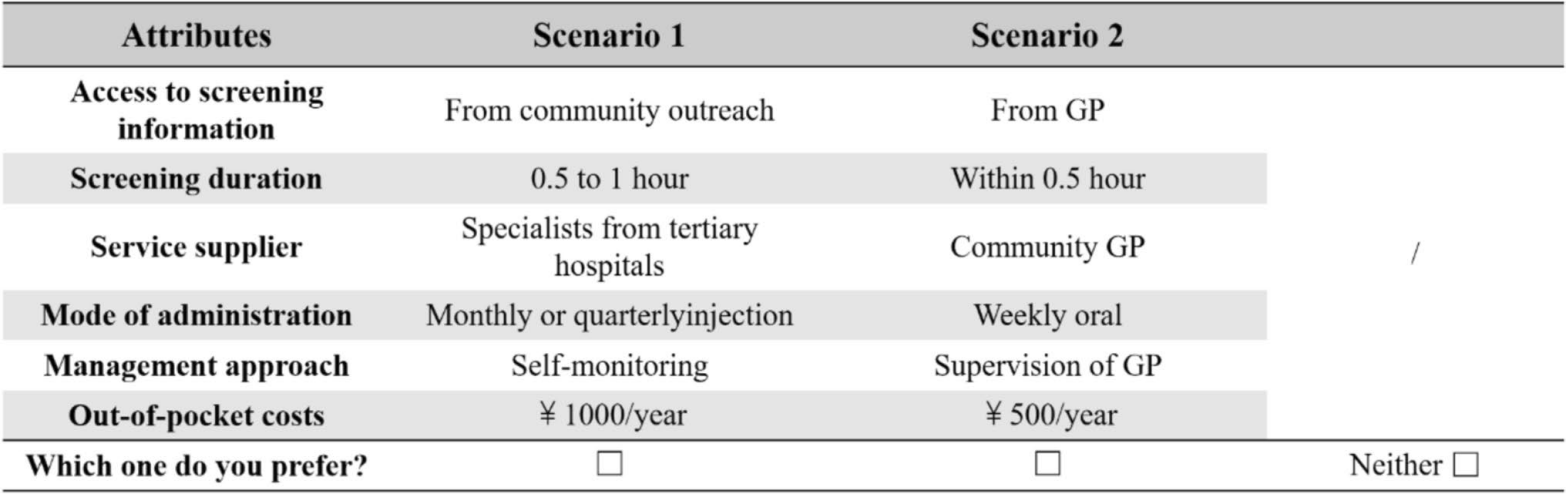
An example question in the discrete choice experiment

The D-optimal design was employed to generate 9 choice sets using SAS (version 9.4; SAS Institute). In DCE studies within healthcare, the number of choice sets typically ranges from 8 to 16. Further “block” design will be required if the number exceeds the above range. The 9 choice sets developed in this study fell within the recommended range and therefore no “block” design was undertaken in this study. A consistency test was included by adding a dominant choice set, in which all the attribute levels in one scenario were clearly superior to those in the other. To better approximate the real-world clinical decision-making, a “Neither” option was also introduced in each set. An illustrative example of a choice set is presented in Fig. 2.

In addition to the 9 choice sets, our questionnaire further incorporated the research background, attribute definitions, and sociodemographic inquiries. A total of 8 factors were included in the final questionnaire: sex, age, education, marital status, annual household income, residence, and presence of osteoporosis. Presence of osteoporosis was determined based on participants’ self-reports rather than bone mineral density tests or medical records. The full questionnaire can be found in Online Appendix.

### Sample selection

The calculation of the sample size was guided by *the rule of Thumb I (Orme, 1998)*, depending on the number of multiple-choice questions, attributes, and levels [24]. The sample size for the DCE main-effects model is calculated as [25]:

$$N>\frac{500c}{t*a}$$

where $c$ denotes the maximum number of levels in any attribute, which is 3 in this study. $t$ is the number of choice sets and was set at 9. $a$ equals 2 in this study, indicating the number of scenarios in each choice set. Based on the above formula, the minimum sample size is 84. Considering the planned sampling of participants from both urban and suburban communities, a target sample of 168 cases was proposed.

### Data collection

Data were collected from 6 communities located in urban and suburban areas of Shanghai through stratified random sampling from 1 June to 30 October, 2023. The prevalence of osteoporosis in Shanghai is high, with 14.2% in men and 21% in women approximately 2010 [26]. Target population was community-dwelling residents aged 50 years and above, rather than patients with diagnosed osteoporosis to reflect broader public preferences. Within each sample community, eligible residents who have lived there for at least 1 year were randomly approached on site. They were randomly invited to finish face-to-face consultations with paper questionnaires and were fully informed of the purpose of the study as well as provided detailed explanations of those attributes and levels. Exclusion criteria include ineligibility, inability to consent the survey, and failure of the consistency test set in our choice sets.

### Pilot testing

We first launched a pilot test with 30 eligible residents of *Community A* in Shanghai. The average questionnaire completion time was 3 min. The coefficient sign and sequence of each attribute in the pilot test were basically consistent with the pre-theoretical assumptions, which verified the rationality of our questionnaire.

### Model specification

The DCE is based on random utility theory. In the scope of this study, respondents’ utility is assumed to be a combination of fixed and random utility. The former is derived mainly from the attributes and demographic characteristics of the respondents, whereas the latter is influenced by other unobserved factors. Each respondent is hypothesized to choose the scenario with the greatest utility. Probability of choosing a given strategy for osteoporosis community management is determined by the indirect utility. And here it is assumed that this is linear and additive and of the form:

$$U={\beta }_{0}+{\beta }_{1}\mathrm{access}+{\beta }_{2}\mathrm{duration}+{\beta }_{3}\mathrm{servicesupplier}+{\beta }_{4}\mathrm{administration}+{\beta }_{5}\mathrm{management}+{\beta }_{6}\mathrm{cost}$$

where ${\beta }_{0}$ represents the baseline coefficient and where ${\beta }_{1}-{\beta }_{6}$ represents the coefficient of each attribute. A mixed logit model was used to analyze the choice data in Stata (version 16.0; StataCorp), and we hypothesized that the parameters of the attributes conformed to a normal distribution. Parameter estimations were derived relative to the reference level within one attribute.

### Attribute relative importance

Relative importance (RI) was used to explore the strength of respondents’ preference for each attribute. Due to the difference in attributes’ reference levels in DCE, relative importance of attributes cannot be compared simply through the regression coefficients. We determined the RI of each attribute by calculating the ratio of the difference between the highest and lowest levels of utility within each attribute to the sum of that difference across all attributes:

$${\mathrm{RI}}_{k}=\frac{{\mathrm{max}\beta }_{k}-{\mathrm{min}\beta }_{k}}{{\sum }_{k=1}^{k}({\mathrm{max}\beta }_{k}-{\mathrm{min}\beta }_{k})}$$

where ${\mathrm{max}\beta }_{k}$ indicates the maximum utility of attribute $k$ and ${\mathrm{min}\beta }_{k}$ is the minimum utility.

### Willingness to pay

Willingness to pay (WTP) was used to measure the amount of money that one respondent would pay to increase a given level of osteoporosis community-level management. We calculated the ratios of the regression coefficients of each attribute to the OOP coefficient, aiming to indicate the relative impact of each attribute on the final choice as the price changes:

$${\mathrm{WTP}}_{x}=-\frac{\partial U/\partial x}{\partial U/\partial \mathrm{cost}}=-\frac{{\beta }_{x}}{{\beta }_{\mathrm{cost}}}$$

where ${\beta }_{x}$ is the regression coefficient for each attribute except the OOP and ${\beta }_{\mathrm{cost}}$ is the coefficient for the OOP.

### Subgroup analyses

Subgroup analyses were conducted by comparing the relative importance of attributes between different groups of respondents, aiming to acquire individual-level preferences. The characteristics of the respondents included age, annual household income, residence, and the presence of osteoporosis.

### Scenario prediction

A useful and necessary output when using DEC to adjust community intervention strategies for osteoporosis is to estimate how the probability of choosing a particular strategy change with different combinations of levels among the attributes. We conducted scenario prediction analysis and applied uptake rate model to predict the probability. The logit probability of choosing alternative strategy $i$ rather than $j$ is given by:

$${P}_{i}=\frac{{e}^{{\beta }_{i}{x}_{i}}}{\sum {e}^{{\beta }_{j}{x}_{j}}}$$

where ${x}_{i}$ and ${x}_{j}$ are the coefficients of attributes in options $i$ and $j$. When the level in this scenario is changed from $i$ to $j$, the possibility of the change chosen $\left({P}_{c}\right)$ is as follows:

$${P}_{c}={P}_{i}-{P}_{j}$$

## Results

### Sample characteristics

A total of 200 questionnaires were collected finally, of which 30 failed the consistency test and were excluded, leaving 170 valid questionnaires for analysis (a completion rate of 85%).

The sociodemographic profile of the 170 respondents is presented in Table 2. The majority of them were female (108/170, 63.53%) and married (148/170, 87.06%), with ages between 71 and 80 years (74/170, 43.53%). Most of them had an education level equivalent to middle school and below (95/170, 55.88%), with an annual household income of less than 50,000 yuan (6993.78 USD). Thirty-three respondents had already been diagnosed with osteoporosis before our survey (33/170, 19.41%).

**Table 2:** Sociodemographic characteristics of 170 participants

Characteristics	Participants, n(%)
Sex	
Male	62(36.47)
Female	108(63.53)
Age	
50–60	25(14.71)
61–70	54(31.76)
71–80	74(43.53)
81 and above	17(10.00)
Education level	
Middle school and below	95(55.88)
High school and above	75(44.12)
Marital status	
Married	148(87.06)
Unmarried and divorced	22(12.94)
Annual household income per year	
50,000 yuan (6993.78 USD) and below	58(34.12)
50,000–100,000 yuan	39(22.94)
100,000 yuan (13987.58 USD) and above	73(42.94)
Geographic location	
Urban	85(50.00)
Suburban	85(50.00)
Already have osteoporosis	
Yes	33(19.41)
No	137(80.59)

### Preferences for osteoporosis community-level management

Table 3 presents the mixed logit model results. Additional results of a conditional logit model are presented in Online Appendix as a supplement **(**Table S1 in Online Appendix).

**Table 3:** Results of the mixed logit model

	*β*^a^	SE	*p* value	SD	SE	*p* value
Access to screening information (reference level when physical examination)						
From community outreach	0.348*	−1.357	0.023	0.153	0.210	0.001
From GP	−0.437*	2.102	0.027	0.197	0.200	< 0.001
Screening duration (reference level within 0.5 h)						
0.5 to 1 h	−1.022***	−0.467	< 0.001	0.108	0.169	0.006
Service supplier (reference level community physician)						
Specialists from tertiary hospitals	0.420**	1.737	0.003	0.140	0.160	< 0.001
Mode of administration (reference level weekly oral)						
Monthly or quarterly injection	−0.244*	0.819	0.031	0.113	0.157	0.001
Management approach (reference level self-monitoring)						
Supervision of GP	0.296*	2.061	0.037	0.142	0.191	0.001
Out-of-pocket costs	−0.002***	−0.002	< 0.001	< 0.001	< 0.001	0.001
Log-likelihood ratio	−1371.583					
*N*	170					
Observations	5100					

^a^*p < 0.05, **p < 0.01, ***p < 0.001

We identified that a total of 6 attributes had a significant effect on respondents’ preferences at the 5% significance level ($\alpha$ = 0.05). The respondents’ utility increased as they acquired screening information from community outreach ($\beta$ = 0.348) compared with access at the site of physical examination, whereas the utility decreased if they knew relative information from GPs ($\beta$ = −0.437). As the screening duration increased to 0.5–1 h, the respondents’ utility declined significantly ($\beta$ = −1.022). They expressed a greater likelihood of choosing specialists from tertiary hospitals than community GPs did ($\beta$ = 0.420). In terms of mode of administration, respondents were more likely to accept weekly oral tablets ($\beta$ = −0.244) than monthly or quarterly injections. In contrast to self-monitoring, the supervision of a GP was obviously more popular among respondents ($\beta$ = 0.296). In addition, their utility showed a downward trend as the OOP decreased, although the decline was relatively slight ($\beta$ = −0.002).

### Relative importance of attributes

**Fig. 3:**
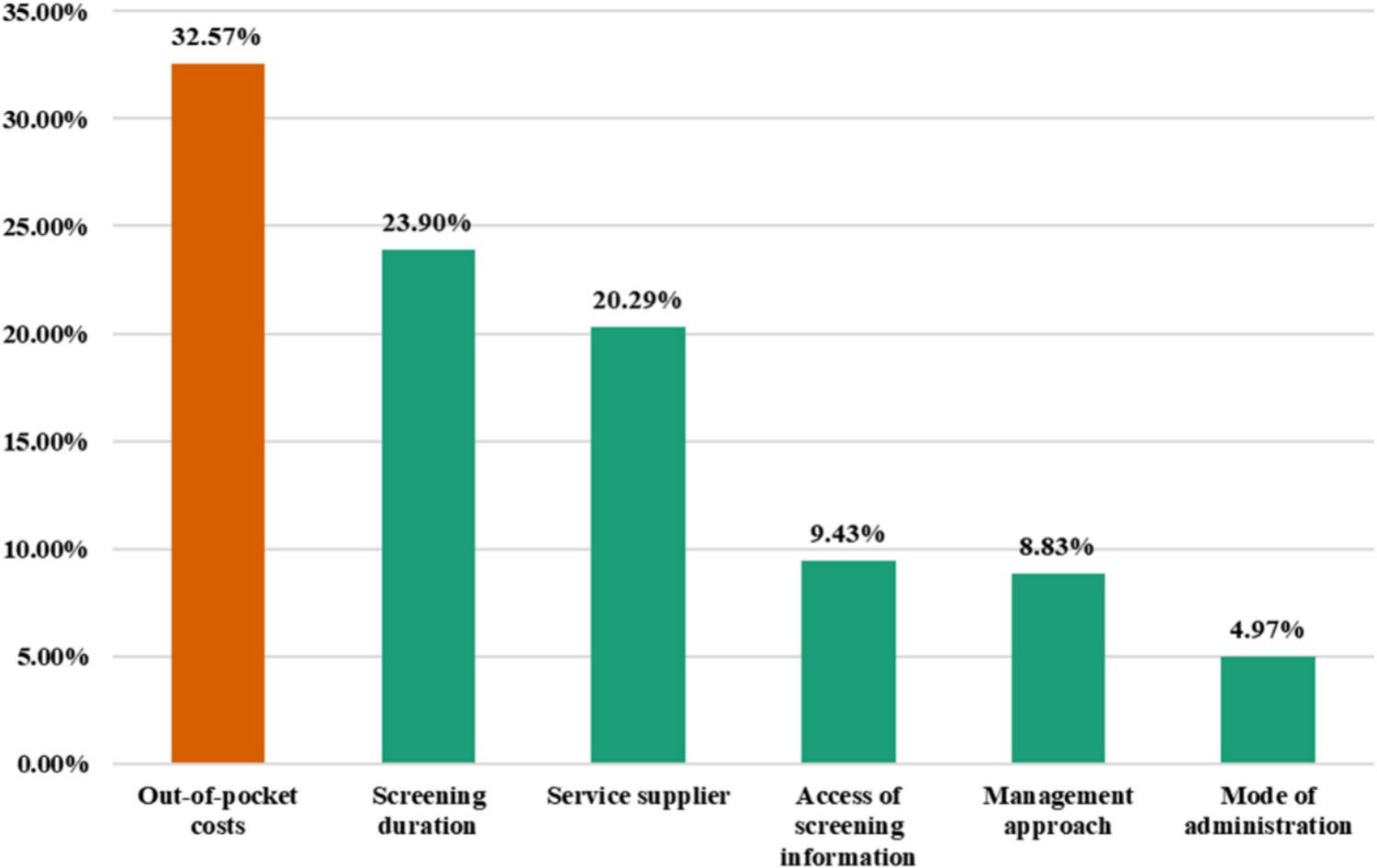
Attributes’ relative importance. Willingness to pay

We further included the OOP as a triple categorical variable in the model, aiming to acquire the attribute relative importance (ARI). Figure 3 shows the results.

OOP emerged as the most significant attribute from the perspective of respondents, with an ARI score of 32.57. The screening duration and service supplier ranked second (ARI = 23.90) and third in importance (ARI = 20.29), respectively. The following attributes were related to access to screening information and the management approach, with ARIs of 9.43 and 8.83, respectively. The mode of administration was considered comparatively not very critical (ARI = 4.97).

**Fig. 4:**
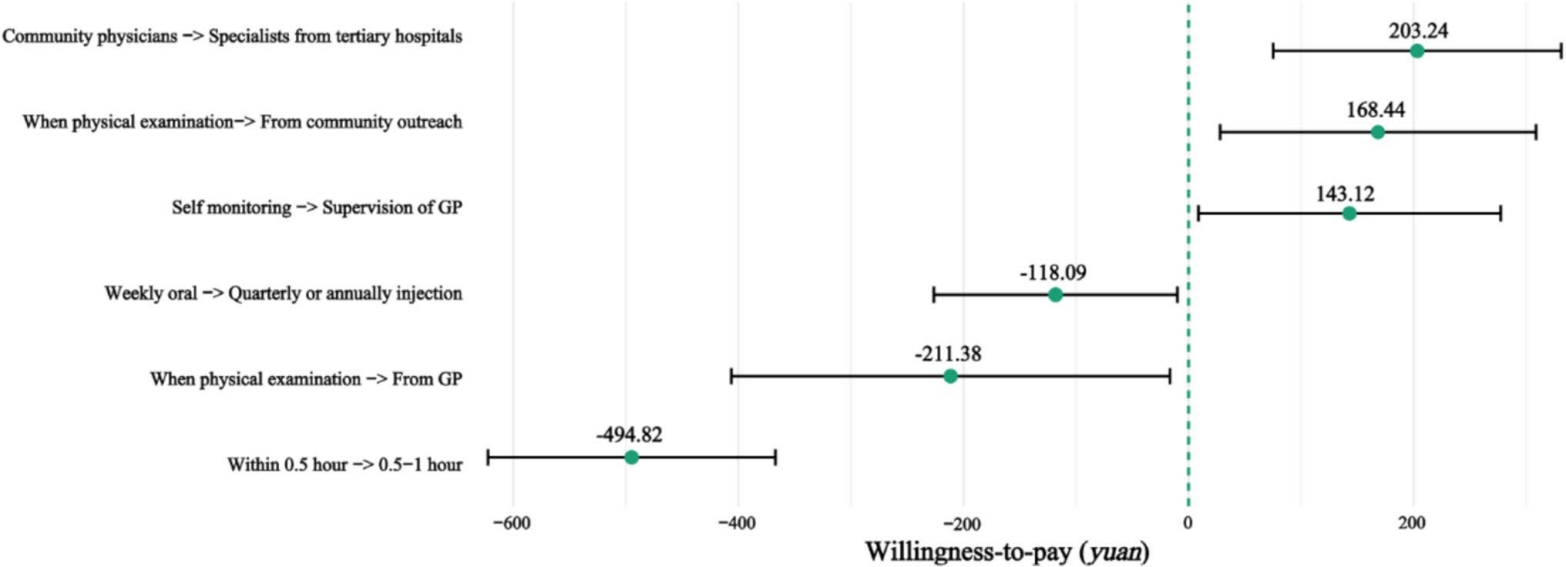
WTP estimates with 95% confidence intervals

We further calculated the willingness to pay (WTP) for each attribute, and the OOP variable is considered a measurement of price. Considering that the coefficient for OOP was slightly small, which may have affected the final WTP results, we used the delta method to process the coefficient in the calculation. Figure 4 presents the WTP results, and detailed results can be found in Table S2 in Online Appendix. As the access to screening information changed to community outreach and GP, the respondents’ WTP increased by 168.44 *yuan* (23.56 *USD*) and decreased by 211.377 *yuan* (29.57 *USD*), respectively, showing opposite directions of influence. When the screening duration was extended to 0.5–1 h, the respondents were willing to pay 494.823 *yuan* (69.22 *USD*) less. The service supplier was likely to afford more than 203.243 *yuan* (28.43 *USD*) for the change from GPs to specialists. Compared with weekly oral tablets, respondents were less likely to choose monthly or quarterly injections, and their WTP decreased by 118.088 *yuan* (16.52 *USD*) when the administration mode changed to the latter. The respondents were willing to pay an extra 143.115 *yuan* (20.02 *USD*) to be supervised by a GP in lieu of self-monitoring.

The WTP of attributes among respondents residing in different areas was also included in our study. The results revealed that urban samples were more willing to pay for access to screening information than respondents who resided in suburban communities, with WTP values of 318.56 *yuan* (44.56 *USD*) and 208.01 *yuan* (29.10 *USD*) for the two levels, respectively. The detailed results can be found in Tables S3 in Online Appendix.

### Subgroup analyses

Subgroup analyses were performed for four sociodemographic variables, including age, annual household income, residence, and presence of osteoporosis. Among them, there was a significant difference between respondents residing in urban or suburban areas (Table S4 in Online Appendix) regarding the effect of each attribute on preferences. The mode of administration and management approach did not significantly affect preferences in either the urban or the suburban samples. Compared with service suppliers, respondents who resided in urban areas placed more importance on screening duration. When the screening duration was extended to 0.5–1 h, their utility presented a more obvious decrease than did the suburban samples. Service suppliers also significantly affected the preferences of those who lived in suburban areas. Those samples expressed stronger preferences for specialists than did community GPs, and the effect was greater than that in urban samples. In terms of access to screening information, urban respondents’ utilities increased at both levels, whereas for those residing in suburban areas, the utilities clearly declined.

### Scenario prediction

We simulated several scenarios with uptake rate models, aiming to calculate the probability that the community population’s choice would change as the attributes changed. On the basis of the mixed logit model results, we set the baseline level at 0 OOP, 0.5–1 h of screening, GPs as service suppliers, access to screening information during physical examination, self-monitoring and weekly oral tablets. We modeled a total of 12 possible scenarios, among which 8 had a single attribute change, while the remaining 4 were multi-attribute change scenarios. The results are presented in Table S5 and Figure S1 in Online Appendix.

As OOP increased to 500 *yuan* (69.94 *USD*) and 1000 *yuan* (139.88 *USD*)*,* the possibility of choosing a service option for osteoporosis community-level management declined by 11.13% and 17.39%, respectively. The possibility increased by 13.60% if the screening duration was limited to 0.5 h. When respondents’ access to screening information changed to community outreach and GPs, the possibility increased by 0.49% and decreased by 1.65%, respectively. The levels adversely affected the expected possibility. Supervision from a GP slightly increased the possibility of choice by 1.13% compared with self-monitoring, whereas monthly or quarterly injection had a reduction of 1.65% compared with weekly oral tablets.

In the 4 multi-attribute change scenarios, we assumed 0 OOP as the baseline level, and the screening duration was set within 0.5 h. Overlaying attribute changes in the management approach and mode of administration on top of this would result in increases of 12.93% and 11.42% in the possibility, respectively. A further change in the service supplier would lead to increases of 18.62% and 15.68% on the basis of the 2 scenarios.

## Discussion

To our knowledge, this is the first study investigating public preferences for osteoporosis community intervention in China. Using data from Shanghai, we examined 6 key attributes closely associated with osteoporosis community intervention. Our findings indicate that respondents placed the greatest importance on OOP costs and screening duration. We further calculated respondents’ WTP for each attribute and simulated several scenarios to predict the possibility that their choice would change as one or more attributes changed.

Respondents were most concerned about OOP costs and screening duration in community intervention strategies in the field of osteoporosis. Their utility decreases as OOP costs increase, which is consistent with prior studies on patients’ preferences for chronic disease treatment [27–29]. Particularly in community intervention, patients tend to favor strategies that reduce the long-term financial burden by minimizing OOP costs. Similarly, respondents’ utility is found to decrease significantly as the screening duration increases. This may be because screening duration is always regarded as a personal time cost [8, 14]. Moreover, community-level screening strategies for osteoporosis are carried out mainly through professional scales and QUS examinations [30]. A longer screening may also imply greater psychological burden and anxiety for people to be screened, especially for elderly individuals [31, 32].

Our findings also reveal that respondents have limited trust in family physicians compared with specialists from tertiary hospitals, despite Shanghai’s substantial efforts in community-level family physician contracting program [33]. Regarding service suppliers, respondents prefer specialists from tertiary hospitals over CHC physicians and family physicians, aligning with previous results on residents’ preferences regarding the content of GP services [29, 31]. While community residents value the role of GPs in providing long prescriptions for chronic diseases, health counseling, and lifestyle guidance [31], limited trust can also be seen through access to screening information [34]. Compared with obtaining information at the site of routine physical examination, respondents’ utility would increase if they acquired through community outreach. This may be related to differences in the reach, route of delivery, and reception scenarios of information. Community outreach is often delivered through brochures, health talks, and other various forms of communication in addition to simple screening information [34, 35]. This may increase residents’ awareness of the importance of screening and motivate them to participate more actively. In addition, community outreach has a wider reach compared to information from GPs, which is typically limited to clinic visits. As a result, residents may not feel urgency because the dissemination of information is limited and more personal in the latter scenario. Previous research also highlights a general lack of awareness among Chinese residents regarding the role of GP in osteoporosis screening, coupled with a preference for systematic chronic disease management [36], which may explain why respondents preferred supervision by GPs instead of self-monitoring*.*

In terms of medication administration, respondents are more likely to accept weekly oral tablets over monthly or quarterly injections. This contrasts with most previous studies, where osteoporosis patients prefer intravenous (IV) infusion over oral infusion and longer medication intervals [21, 23]. However, some studies have also indicated that older patients prefer oral tablets due to the fear of the invasive treatments [19]. While IV means a longer treatment interval, it requires professional medical operations, whereas weekly oral medication may support regular self-management habits [33]. Moreover, our sample included non-patients, whose perceptions of IV treatment’s long-term effects may be underestimated due to the absence of direct health impact.

Significant heterogeneity has also been found between urban and suburban residents in preferences for osteoporosis community intervention. For instance, receiving screening information from GPs increases the utility among urban residents but decreases it among suburban samples. This difference likely stems from disparities in GP availability between urban and suburban Shanghai [35, 37]. Suburban area have fewer GPs and more dispersed populations, making GP service provision less convenient and reducing trust levels [31].

Overall, our findings highlight that GPs undoubtedly play a major role in community interventions for osteoporosis, although residents’ trust varies based on their expectations of GP roles. While trust in GPs to provide screening information seems relatively low, their participation in long-term supervision and follow-up has been well recognized. Shanghai can enhance GPs’ expertise through specialty training focused on chronic disease management to strengthen community intervention effect on osteoporosis. In addition, as the primary screening sites, CHCs can further improve screening equipment and integrate medical technology to boost osteoporosis prevention efforts.

## Limitations

Our study still has some limitations. First, the DCE was deliberately scoped to modifiable, community-level features. We estimated a main-effects mixed logit model without interaction terms, which may contribute to unobserved heterogeneity. Second, the analytic sample comprised 170 community-dwelling adults recruited on site from 3 urban and 3 suburban communities in Shanghai using stratified design with convenience recruitment. As a result, subgroup comparisons were exploratory and per-group sizes were relatively small. Future studies should recruit larger, prospectively powered samples across more communities to improve precision, enable confirmatory subgroup analyses. Besides, because the data were collected in Shanghai, a setting with a relatively older population structure and a well-developed community health service system, generalization to other regions of China should be made with caution.

## Conclusion

Our study significantly contributes to understanding of residents’ preferences for osteoporosis community-level management. Acknowledging the impact of attributes in community-level strategies on residents’ utility and willingness to pay is important for optimizing osteoporosis community-level strategies. It is necessary and useful to further promote early screening, diagnosis, and treatment of osteoporosis.

## Supplementary Information

Below is the link to the electronic supplementary material.

Supplementary file1 (DOCX 131 KB)

Supplementary file2 The supplementary material includes the questionnaire, results of the conditional logit model, WTP, mixed logit model, WTP calculated by urban respondent’s vs suburban respondents and scenario prediction results. (DOCX 25 KB)

## Data Availability

The datasets used and/or analyzed during the current study are available from the corresponding author on reasonable request. The authors will supply the relevant data in response to reasonable requests.

## Abbreviations

DCE: Discrete experiment choice
CHC: Community health center
OOP: Out-of-pocket
WTP: Willingness to pay
GP: General practitioner
