# Supplementary material for: Using discrete choice experiment to investigate public preferences for osteoporosis community-level management strategies in China
Source: J Bone Miner Metab. 2025 Nov 21;44(1):58–68. doi: 10.1007/s00774-025-01659-y (PMC12891145; doi:10.1007/s00774-025-01659-y)
Supplement: Supplementary file 1 — Supplementary file1 (DOCX 131 KB) [file 774_2025_1659_MOESM1_ESM.docx]

**Table S1. Conditional logit model results.**

|  | **β**^a^ | **SE** | ***P* value** | **[95% conf. interval]** | |
| --- | --- | --- | --- | --- | --- |
| **Access to screening information *(reference level when physical examination)*** |  |  |  |  |  |
| From community outreach | 0.298** | 0.093 | 0.001 | [0.115, | 0.481] |
| From GP | 0.232** | 0.089 | 0.009 | [0.057, | 0.407] |
| **Screening duration *(reference level within 0.5 hour)*** |  |  |  |  |  |
| 0.5 to 1 hour | -0.236** | 0.076 | 0.002 | [-0.386, | -0.087] |
| **Service supplier *(reference level community physician)*** |  |  |  |  |  |
| Specialists from tertiary hospitals | 0.951*** | 0.070 | <0.001 | [0.814, | 1.088] |
| **Mode of administration *(reference level weekly oral)*** |  |  |  |  |  |
| Monthly or quarterly injection | -0.123 | 0.078 | 0.111 | [-0.276, | 0.029] |
| **Management approach *(reference level self-monitoring)*** |  |  |  |  |  |
| Supervision of GP | 0.311*** | 0.075 | <0.001 | [0.164, | 0.459] |
| **Out-of-pocket costs** | -0.001*** | 0.001 | <0.001 | [-0.001, | -0.002] |
|  |  |  |  |  |  |
| Log-likelihood ratio | -2745.23 | | | | |
| N | 170 | | | | |
| Observations | 5100 | | | | |

^a^: ** p < 0.05，** p < 0.01，*** p < 0.001;*

**Table S2. WTP results.**

|  | **WTP, *yuan****^a^* | **[95% conf. interval]** | |
| --- | --- | --- | --- |
| **Access to screening information** |  |  |  |
| When physical examination→From community outreach | 168.440 | [28.122, | 308.757] |
| When physical examination → From GP | -211.377 | [-406.303, | -16.450] |
| **Screening duration** |  |  |  |
| Within 0.5 hour → 0.5 to 1 hour | -494.823 | [-622.579, | -367.068] |
| **Service supplier** |  |  |  |
| Community Physicians → Specialists from tertiary hospitals | 203.243 | [75.252, | 331.234] |
| **Mode of administration** |  |  |  |
| Weekly oral → Monthly or quarterly injection | -118.088 | [-226.252, | -9.925] |
| **Management approach** |  |  |  |
| Self-monitoring → Supervision of GP | 143.115 | [8.755, | 277.474] |

^a^: 1 yuan= 0.1398 USD

**Table S3. WTP results for samples from urban and suburban areas.**

|  | **Urban, n=85** | | | **Suburban, n=85** | | |
| --- | --- | --- | --- | --- | --- | --- |
|  | **WTP, *yuan****^a^* | **[95% conf. interval]** | | **WTP, *yuan*** | **[95% conf. interval]** | |
| **Access to screening information** |  |  |  |  |  |  |
| When physical examination →From community outreach | 318.557 | [150.189, | 486.825] | -23.379 | [-229.726, | 183.069] |
| When physical examination→From GP | 208.011 | [-1.265, | 417.288] | -213.967 | [-483.805, | 10.873] |
| **Screening duration** |  |  |  |  |  |  |
| Within 0.5 hour → 0.5 to 1 hour | -502.035 | [-677.677, | 326.393] | -290.820 | [-444.119, | -137.521] |
| **Service supplier** |  |  |  |  |  |  |
| Community physicians → Specialists from tertiary hospitals | 379.817 | [191.398, | 568.235] | 309.068 | [188.332, | 429.803] |
| **Mode of administration** |  |  |  |  |  |  |
| Weekly oral → Monthly or quarterly injection | -132.231 | [-324.308, | 59.845] | -13.879 | [-15.097, | 87.339] |
| **Management approach** |  |  |  |  |  |  |
| Self-monitoring → Supervision of GP | 158.675 | [-31.065, | 348.414] | 52.848 | [-72.831, | 178.527] |

^a^: 1 yuan= 0.1398 USD

**Table S4. Mixed logit model results for samples from urban areas and suburban areas.**

|  | **Urban, n=85** | | **Suburban, n=85** | |
| --- | --- | --- | --- | --- |
|  | **β**^a^**(SE)** | **SD (SE)** | **β(SE)** | **SD (SE)** |
| **Access to screening information *(reference level when physical examination)*** |  |  |  |  |
| From community outreach | 0.645***(0.179) | -0.656***(0.244) | -0.070(0.314) | 2.528***(0.499) |
| From GP | 0.421*(0.222) | 1.576***(0.268) | -0.642*(0.343) | 2.831***(0.391) |
| **Screening duration *(reference level within 0.5 hour)*** |  |  |  |  |
| 0.5 to 1 hour | -1.017***(0.139) | 0.387**(0.180) | -0.873***(0.231) | -1.516***(0.301) |
| **Service supplier *(reference level community physician)*** |  |  |  |  |
| Specialists from tertiary hospitals | 0.769***(0.194) | 1.674***(0.248) | 0.927***(0.189) | 1.005***(0.210) |
| **Mode of administration *(reference level weekly oral)*** |  |  |  |  |
| Monthly or quarterly injection | -0.268(0.188) | 1.440***(0.271) | -0.042(0.155) | -0.268(0.252) |
| **Management approach *(reference level self-monitoring)*** |  |  |  |  |
| Supervision of GP | 0.321(0.199) | 2.013***(0.232) | 0.159(0.193) | 1.221***(0.185) |
| **Out-of-pocket costs** | -0.002***(<0.001) | -0.002***(<0.001) | -0.003***(<0.001) | 0.002***(<0.001) |

^a^: ** p < 0.05，** p < 0.01，*** p < 0.001;*

**Table S5. Scenario prediction results.**

| **number** | **OOP** | **Screening duration** | **Service supplier** | **Access to screening information** | **Management approach** | **Mode of administration** | **P change (%)** | **95% CI (%)** |
| --- | --- | --- | --- | --- | --- | --- | --- | --- |
| **baseline** | **0** | **0.5-1 hour** | **Community physicians** | **When physical examination** | **Self-monitoring** | **Weekly oral** | **/** | **/** |
| (1) | 500 | 0.5-1 hour | Community physicians | When physical examination | Self-monitoring | Weekly oral | -11.13 | (-18.55, -6.49) |
| (2) | 1000 | 0.5-1 hour | Community physicians | When physical examination | Self-monitoring | Weekly oral | -17.39 | (-30.23, -7.59) |
| (3) | 0 | Within 0.5 hour | Community physicians | When physical examination | Self-monitoring | Weekly oral | 13.60 | (9.80, 17.75) |
| (4) | 0 | 0.5-1 hour | Specialists | When physical examination | Self-monitoring | Weekly oral | 8.69 | (2.91, 14.41) |
| (5) | 0 | 0.5-1 hour | Community physicians | From community outreach | Self-monitoring | Weekly oral | 0.49 | (-12.92, 12.81) |
| (6) | 0 | 0.5-1 hour | Community physicians | From GP | Self-monitoring | Weekly oral | -1.65 | (-8.44, 5.89) |
| (7) | 0 | 0.5-1 hour | Community physicians | When physical examination | Supervision of GP | Weekly oral | 1.13 | (-5.53, 7.65) |
| (8) | 0 | 0.5-1 hour | Community physicians | When physical examination | Self-monitoring | Monthly/ quarterly injection | -1.72 | (-5.94,2.55) |
| (9) | 0 | Within 0.5 hour | Community physicians | When physical examination | Supervision of GP | Weekly oral | 12.93 | (1.52, 21.35) |
| (10) | 0 | Within 0.5 hour | Community physicians | When physical examination | Self-monitoring | Monthly/ quarterly injection | 11.42 | (5.81, 17.11) |
| (11) | 0 | Within 0.5 hour | Specialists | When physical examination | Supervision of GP | Weekly oral | 18.62 | (1.93, 31.21) |
| (12) | 0 | Within 0.5 hour | Specialists | When physical examination | Self-monitoring | Monthly/ quarterly injection | 15.68 | (2.53, 28.56) |

**Figure S1. Uptake rate for scenario prediction.**

**
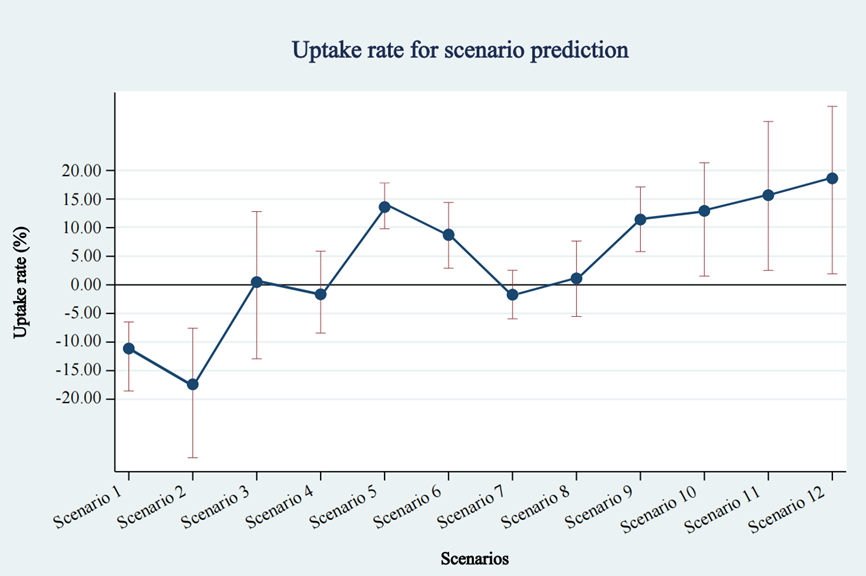
**
