# Supplementary material for: Using discrete choice experiment to investigate public preferences for osteoporosis community-level management strategies in China
Source: J Bone Miner Metab. 2025 Nov 21;44(1):58–68. doi: 10.1007/s00774-025-01659-y (PMC12891145; doi:10.1007/s00774-025-01659-y)
Supplement: Supplementary file 2 — Supplementary file2 The supplementary material includes the questionnaire, results of the conditional logit model, WTP, mixed logit model, WTP calculated by urban respondent’s vs suburban respondents and scenario prediction results. (DOCX 25 KB) [file 774_2025_1659_MOESM2_ESM.docx]

**Questionnaire on preferences for osteoporosis community-level management strategies**

***Number：_______ ID：_____________***

Dear respondents！ We are researchers at the *School of Public Health, Fudan University*. Currently we are conducting a questionnaire survey on the topic titled ***Public preferences for osteoporosis community-level management strategies in China***. The information you provide is only for the purpose of conducting the project, and we will keep your answers confidential, so please fill in the questionnaire truthfully!

- **Part 1: Personal Information**

1. Your gender is:
2. Male；
3. Female；
4. Your age is _____;
5. Your education level is:
6. Primary school and below;
7. Junior high school;
8. Senior high school;
9. Bachelor's degree and above.
10. Your marital status：
11. Married;
12. Unmarried;
13. Divorced;
14. Your annual household income is ___________ yuan
15. whether you have lived in the community for 1 year or more:
16. Yes;
17. No;
18. whether you are currently suffering from osteoporosis:
19. Yes;
20. No;
21. whether you currently suffer from any underlying disease other than osteoporosis (e.g., high blood pressure, diabetes.):
22. Yes; the disease _________________;
23. No;

- **Part 2: Scenario Selection**

**Please select your favorite answer for each of the following 10 choice sets in order**

- **SET 1**

| **Attributes** | **Scenario 1** | **Scenario 2** |  |
| --- | --- | --- | --- |
| Access to screening information | From community outreach | From GP | / |
| Screening duration | 0.5-1 hour | Within 0.5 hour |  |
| Service supplier | Specialists from tertiary hospitals | Community GP |  |
| Mode of administration | Monthly or quarterly injection | Weekly oral |  |
| Management approach | Self-monitoring | Supervision of GP |  |
| Out-of-pocket costs | ￥1000/year | ￥500/year |  |
| Which one do you prefer? | □ | □ | Neither□ |

- **SET 2**

| **Attributes** | **Scenario 1** | **Scenario 2** |  |
| --- | --- | --- | --- |
| Access to screening information | From community outreach | When physical examination | / |
| Screening duration | Within 0.5 hour | 0.5-1 hour |  |
| Service supplier | Specialists from tertiary hospitals | Community GP |  |
| Mode of administration | Weekly oral | Monthly or quarterly injection |  |
| Management approach | Self-monitoring | Supervision of GP |  |
| Out-of-pocket costs | ￥500/year | ￥1000/year |  |
| Which one do you prefer? | □ | □ | Neither□ |

- **SET 3**

| **Attributes** | **Scenario 1** | **Scenario 2** |  |
| --- | --- | --- | --- |
| Access to screening information | From GP | When physical examination | / |
| Screening duration | 0.5-1 hour | Within 0.5 hour |  |
| Service supplier | Specialists from tertiary hospitals | Community GP |  |
| Mode of administration | Weekly oral | Monthly or quarterly injection |  |
| Management approach | Supervision of GP | Self-monitoring |  |
| Out-of-pocket costs | None | ￥500/year |  |
| Which one do you prefer? | □ | □ | Neither□ |

- **SET 4**

| **Attributes** | **Scenario 1** | **Scenario 2** |  |
| --- | --- | --- | --- |
| Access to screening information | From GP | When physical examination | / |
| Screening duration | 0.5-1 hour | Within 0.5 hour |  |
| Service supplier | Community GP | Specialists from tertiary hospitals |  |
| Mode of administration | Weekly oral | Monthly or quarterly injection |  |
| Management approach | Self-monitoring | Supervision of GP |  |
| Out-of-pocket costs | ￥1000/year | None |  |
| Which one do you prefer? | □ | □ | Neither□ |

- **SET 5**

| **Attributes** | **Scenario 1** | **Scenario 2** |  |
| --- | --- | --- | --- |
| Access to screening information | When physical examination | From GP | / |
| Screening duration | 0.5-1 hour | Within 0.5 hour |  |
| Service supplier | Community GP | Specialists from tertiary hospitals |  |
| Mode of administration | Weekly oral | Monthly or quarterly injection |  |
| Management approach | Self-monitoring | Supervision of GP |  |
| Out-of-pocket costs | None | ￥1000/year |  |
| Which one do you prefer? | □ | □ | Neither□ |

- **SET 6**

| **Attributes** | **Scenario 1** | **Scenario 2** |  |
| --- | --- | --- | --- |
| Access to screening information | From GP | From community outreach | / |
| Screening duration | 0.5-1 hour | Within 0.5 hour |  |
| Service supplier | Specialists from tertiary hospitals | Community GP |  |
| Mode of administration | Monthly or quarterly injection | Weekly oral |  |
| Management approach | Self-monitoring | Supervision of GP |  |
| Out-of-pocket costs | ￥500/year | ￥1000/year |  |
| Which one do you prefer? | □ | □ | Neither□ |

- **SET 7**

| **Attributes** | **Scenario 1** | **Scenario 2** |  |
| --- | --- | --- | --- |
| Access to screening information | When physical examination | From community outreach | / |
| Screening duration | Within 0.5 hour | 0.5-1 hour |  |
| Service supplier | Specialists from tertiary hospitals | Community GP |  |
| Mode of administration | Weekly oral | Monthly or quarterly injection |  |
| Management approach | Self-monitoring | Supervision of GP |  |
| Out-of-pocket costs | ￥1000/year | ￥500/year |  |
| Which one do you prefer? | □ | □ | Neither□ |

- **SET 8**

| **Attributes** | **Scenario 1** | **Scenario 2** |  |
| --- | --- | --- | --- |
| Access to screening information | From community outreach | When physical examination | / |
| Screening duration | Within 0.5 hour | 0.5-1 hour |  |
| Service supplier | Community GP | Specialists from tertiary hospitals |  |
| Mode of administration | Monthly or quarterly injection | Weekly oral |  |
| Management approach | Self-monitoring | Supervision of GP |  |
| Out-of-pocket costs | None | ￥500/year |  |
| Which one do you prefer? | □ | □ | Neither□ |

- **SET 9**

| **Attributes** | **Scenario 1** | **Scenario 2** |  |
| --- | --- | --- | --- |
| Access to screening information | From community outreach | When physical examination | / |
| Screening duration | 0.5-1 hour | Within 0.5 hour |  |
| Service supplier | Community GP | Specialists from tertiary hospitals |  |
| Mode of administration | Weekly oral | Monthly or quarterly injection |  |
| Management approach | Supervision of GP | Self-monitoring |  |
| Out-of-pocket costs | ￥500/year | None |  |
| Which one do you prefer? | □ | □ | Neither□ |

- **SET 10**

| **Attributes** | **Scenario 1** | **Scenario 2** |  |
| --- | --- | --- | --- |
| Access to screening information | From GP | When physical examination | / |
| Screening duration | 0.5-1 hour | Within 0.5 hour |  |
| Service supplier | Community GP | Specialists from tertiary hospitals |  |
| Mode of administration | Weekly oral | Weekly oral |  |
| Management approach | Supervision of GP | Supervision of GP |  |
| Out-of-pocket costs | ￥1000/year | None |  |
| Which one do you prefer? | □ | □ | Neither□ |
